# Clinical nursing competency during epidemics: a qualitative content analysis

**DOI:** 10.1186/s12912-024-01977-y

**Published:** 2024-05-03

**Authors:** Leila Abadian, Negin Masoudi Alavi, Zahra Tagharrobi

**Affiliations:** Trauma Nursing Research Center, Faculty of Nursing, Kashan University of Medical Sciences, Kashan, Iran

**Keywords:** Clinical competence, Nurses, Epidemic, Concept analysis

## Abstract

**Background:**

Nurses are on the frontline for managing epidemic diseases. Different aspects of clinical nursing competencies during epidemics are important issues that need investigation.

**Objectives:**

The aim of this study was to determine the required clinical competencies for nurses during epidemics. Understanding these competencies could provide valuable information for health care services and nursing education organizations to prepare nurses for future epidemics.

**Methods:**

The qualitative conventional content analysis study was conducted using semi-structured interviews with 12 nurses that were actively engaged in providing patient care during COVID-19 pandemic in Shahid Beheshti hospital in Kashan/Iran, from October 2022 to March 2023. The data analysis process was conducted according to 5 steps suggested by Graneheim and Lundman.

**Results:**

After analysis, the 159 competencies were derived from interviews that were categorized to 11 subcategories, and three categories of clinical nursing skills in epidemics, knowledge of epidemics, and soft skills for nurses in epidemics.

**Conclusion:**

Nurses need wide range of competencies to address the professional expectations regarding providing acceptable care during epidemics. Knowing these competencies can help nursing managers to prepare nurses for crisis such as what world experienced during COVID-19 pandemic.

**Supplementary Information:**

The online version contains supplementary material available at 10.1186/s12912-024-01977-y.

## Introduction

Infectious diseases outbreaks are threatening people’s physical, mental, and social health. They also disrupt the economic, cultural, and political balance of the society. Nurses play a major role in controlling and managing infection outbreaks [1]. The studies have shown that high quality nursing care could decrease the rate of mortality during epidemics by 70% [2].

Competency is an ability that is achieved through experience and learning. International Council of Nurses has defined the main competencies of nursing as the ability to make the right decision at critical times, protecting safety, and appropriate clinical skills [3]. Nurses need special competencies to ensure their quality of care in epidemics. Previous studies are mostly about the competencies of nurses in routine situations. Providing patient-oriented care, being professional, using technology and informatics, evidence-based functioning, leadership, having communication skills, and teamwork ability are the main competencies in routine nursing care [4]. Few studies have been conducted about nurses’ competencies during a crisis. The six core competencies of nurses in crisis and disasters management have been defined as: task and resource management, situational awareness, teamwork, communication, control of emotions, and leadership [5]. The ability of the nurse to recognize signs of clinical deterioration, interpret the assessment data, and arrive to sound clinical judgement promptly is considered a cornerstone of managing patients in crisis [6]. Competencies of nurses in routine situations, and the specific competencies during crisis are important but cannot cover all the aspects of nurses’ job demands during outbreaks of infectious diseases. As was experienced during COVID-19 pandemic, there would be overload of patients in emergency wards and ICUs, and considerable shortages in staff, space and equipment [7]. Nurses should provide a high quality care for the patients in spite of these shortages, and on the other hand, overcome their own concerns and fears. They should take care of critical patients under tough circumstances while using personal protective devices. Nurses have stated that they felt like being in the warzone [8]. .

In epidemics, nurses need more competencies, such as knowledge of infectious diseases, and using personal protective equipment correctly [9]. In a qualitative study, the nursing students noted that they should possess the competencies of managing emotional experiences, implementing strategies to control and prevent infections, and the ability to participate in social anti-epidemic activities during COVID-19 epidemic [10] Another study showed that the nurses’ competencies of interpersonal relationships and desire to doing research were improved during the COVID-19 outbreak [11]. Lack of adequate experience and education during biological crises, are the main obstacle for providing effective care [12]. During COVID-19 pandemic, nurses faced a situation that they had never experienced before in terms of intensity and duration, and their competencies in providing quality care were challenged. The competencies of nurses during epidemics, and their experiences in this area have not been fully investigated. Determining these aspects could provide valuable information for the authorities and educators to prepare nurses for future epidemics. The present study was a qualitative conventional content analysis study that was conducted to determine the required clinical competencies of nurses during epidemics.

## Methods

### Research design

This study used a qualitative approach with conventional content analysis. This method is generally used to describe a concept or phenomenon, when existing theory or research literature on the subject is limited. In conventional content analysis, codes, categories and subcategories are obtained directly from interviews or group discussions [13]. The researchers wanted to qualitatively study the experiences of the nurses during COVID-19 pandemic to identify the competencies that nurses should have to successfully manage patients during epidemics when there is a chaotic situation.

### Research participants and settings

Participants were nurses who were working for at least 6 months during the COVID-19 pandemic and agreed to share their experiences. The purposive sampling was used to select nurses with various positions and experiences. To have the highest diversity, researchers tried to invite participants from different wards, and from both genders. In addition, nurses who had significant performance during the pandemic according to the colleagues were invited for the study. For example, the first participant was a 27-year-old nurse with 4 years’ clinical experience, who volunteered to work in COVID-19 ward from the beginning to the end of the pandemic. The second participant was 46-year-old nurse with 17 years’ clinical experience in an emergency ward who, according to her colleagues, was the main supporter and leader of the younger nurses during the pandemic. All the nurses were working in Shahid Beheshti hospital of Kashan/Iran, which is the only general governmental hospital in Kashan Province, with 740 beds that provides health care services to 400,000 residents. During the pandemic, this hospital was the center for providing services to COVID-19 patients. This study was carried out from October 2022 to March 2023.

### Data collection

Data was collected through individual semi-structured in-depth interviews, supported by field notes. All the interviews were conducted by the first author. First, she made a telephone call to the nurses and introduced herself, and arranged for a face-to-face interview. The participants were informed about the objective of the study and informed consent was obtained along with the permission to audio record the interviews. The interviews were carried out in the silent room in the hospital at the times that were appropriate for participants. The interviews included open questions. The nurses were asked about their experiences during COVID-19 pandemic. The key questions were as follows:

- Would you please explain one of your work days during the COVID-19 pandemic?
- What clinical competencies helped you during the pandemic to provide better care for patients?
- Explain your experiences of caring for patients during the pandemic?
- What problems did you experience as a nurse during the pandemic?
- Can you talk about your colleagues that were known as very good and successful nurses during pandemic? Why do you think they were iconic? Can you give an example?

The probing questions were used during interviews to obtain more information and resolve the ambiguity in the information provided.

### Data analysis and trustworthiness

The demographics of participants including sex, age, work experience, marriage, and hospital departments were recorded and analyzed descriptively. The qualitative data analysis process was conducted according to 5 steps suggested by Graneheim and Lundman [14]. In the first step, the first and second authors listened to the recorded interviews several times. Later, the interviews were transcribed by the first author. In the second step, the texts of the interviews were read several times to distinguish meaning units that were parts of the interviews that had a specific message related to the subject (the indicators related to nursing clinical competencies in epidemics). In the third step, texts were read word by word, and initial codes were retrieved. In the fourth step, the codes were categorized based on similarities and relations in abstract classes. In the final step the main conceptual categories and sub-categories were formed. An example of data analysis can be seen in Table 1. For data management, MAXQDA 20 software was used.

To confirm the trustworthiness of the study, Lincoln and Guba’s criteria were used, including credibility, dependability, confirmability, and transferability [15]. Data credibility was achieved by prolonged engagement of the researchers with the subject, using 2 pilot interviews, checking the interviews and analysis by some participants, and frequent debriefing session with project members. The dependability of the study was assured by developing a detailed track record of the data collection process, and measuring coding accuracy by the third author. The confirmability was assured by implementing reflexive weekly meetings of the investigators. The transferability was achieved by inviting participants with a variety of experiences using purposive sampling, and data saturation.

**Table 1:** An example of data analysis

Meaning Unit	Open Code	Sub-categories	Categories
The streets were all deserted. *Every day on the news it was said that this doctor or that nurse had died of COVID.* The family would take the patient to the hospital and run away. Whenever I went to the hospital, I thought I might not come back home. You must *have a strong spirit* to be able to work.	Working in scary conditions	Resilience and endurance	Soft skills for nurses in epidemics
	Having strong spirit	Self-actualization	
The *drugs were unknown, and we had not used them*. *We did not know what side effects they had.* Many of them later turned out to be ineffective, or their side effects were worse. For example, my patient, who is diabetic, does this medicine interact with his other medicines or not? *We had to study and search regularly.*	Ability to administer new drugs	General nursing skills	Clinical nursing skills in epidemics
	Searching new drugs	Being update	Knowledge of epidemics
	Monitor patients for drug side effects	General nursing skills	Clinical nursing skills in epidemics

### Ethical considerations

The present study was approved by the ethics committee of the Kashan University of Medical Sciences under the ethics code of IR.KAUMS.NUHEPM.REC.1401.030. Before starting the study, the goals of the study were explained for the participants. Also, the participants were assured that their data would remain confidential and would only be used regarding the goals of the study. Informed consent was obtained from the participants. Interviews were conducted based on the time schedule of the participants. The Helsinki declaration was respected in this study.

## Results

The interviews were conducted with 12 nurses (8 females and 4 males). The mean age of the participants was 32.75 ± 7.74 years (Range 26–47) and their work experience was 8.83 ± 4.8 years (Range 4–18). The 10 participants were clinical nurses (from intensive care units, emergency, internal, surgical and pediatric departments), 1 was matron and 1 was supervisor. Eight nurses were married. The duration of interviews was between 35 and 60 min. After 10 interviews, no new code was extracted, but interviews continued with 2 more participants to assure data saturation.

After analysis, the 159 competencies were derived from interviews that were categorized to 11 subcategories, and 3 categories (Table 2):

**Table 2:** Categories and subcategories of clinical nursing competency in epidemics

**Categories**	**Subcategories**	**Examples of the abilities**
**Clinical nursing skills in epidemics**	Respiratory system management skills	Ability to work with ventilator, CPAP, ECMO, ability to intubate the patient, chest physiotherapy skill, distinguishing respiratory sounds
	General nursing skills	Patient examination, medicinal treatment, advanced CPR
	Infection control skills	Isolation, disinfection, using personal protective devices
	Ability to work in multiple departments	Working at intensive care units, working at emergency wards, management of critical patients, triage
**Knowledge of epidemics**	Being update	Self-educating, participating in constant educational programs
	Knowledge of infectious diseases	Knowing antibiotics and antivirals, Practical knowledge about the cycle of infection
**Soft skills for nurses in epidemics**	Internal and external professional communication	Cooperation, supporting young nurses, cooperation with the medical team
	Crisis management and leadership	Ability to start a new ward, conflict management, problem solving, critical thinking, organizational, accountability
	Resilience and endurance	Stress management, high compatibility, Ability to work in difficult conditions, conflict and anger management, adaptability
	Self-actualization	Courage, altruism, positive thinking, self-confidence, high internal motivation
	Creativity and artistic vision	Having creativity, using art in nursing

- Clinical nursing skills in epidemics.
- Knowledge of epidemics.
- Soft skills for nurses in epidemics.

### Clinical nursing skills in epidemics

Clinical nursing skills means, the psychomotor, and technical abilities of nurses to successfully manage patients during epidemics. The participants mentioned wide range of clinical competencies that made the subcategories of: respiratory system management skills, general nursing skills, infection control skills, and ability to work in multiple departments.

The skills of intubation, working with devices such as ventilators, CPAP, Bipap, extracorporeal membrane oxygenation (ECMO), and doing postural drainage were some respiratory system management skills that nurses should have to work efficiently in respiratory infection outbreaks. A 32 male nurse stated: *“During corona, there were times that several patients needed intubation at the same time. A nurse should have the capability to manage airway by himself, you couldn’t expect help from physicians and other more experienced colleagues.”*

The patient assessment, hand hygiene, documentation, IV therapy, and drug administration were some general nursing skills that helped nurses to manage epidemics. A 47-year-old male nurse said: *“Our experienced nurses were very helpful, I remember we had a patient, that only Mr.…. could find his veins for IV therapy. One time he inserted a green cannula to the jugular vein.”*

Effective disinfection, wearing personal protective devices, patient isolation, and managing infectious deceased patients were some skills of infection control. The ability to work in multiple departments seems to be crucial for nurses during epidemics, according to the interviews. A 28 years old female nurse stated that: *“I think it is very important that all the nurses rotate in different wards. When someone has worked for 20 or 14 years in surgical or internal wards and all of a sudden she/he has to work in COVID-19 department, it is clear that she cannot handle it.”* Another participant stated that: “*During COVID pandemic, nurses who had worked at different wards were more successful. Well, I have learned a lot during the pandemic, it was like 5 or 6 years of clinical experience.”*

### Knowledge of epidemics

Nurses should have an acceptable knowledge about infectious diseases, and epidemiology principals. More important, when managing little-known infectious diseases, they should update their information, and provide an evidence based nursing care. The sub-categories of being update, and knowledge of infectious diseases made this category. At the beginning of the epidemics, the knowledge about the transmission, the incubation period, the effective treatments, and the complications were limited. So the health care team, including nurses, should have updated their knowledge about the disease.

In this regard, one of the participants stated: *“We were in the middle of the emergency ward and had no information about the disease. We had to try to find new information about it, so that we could provide care for the patients.”* Another participant said: *“In the beginning of the COVID there were many misconceptions about the disease and its transfer and treatment. Patients were continuously asking questions. Nurses should have reliable information to help people.”* The appropriate knowledge about the microbiology, the ways of transmission and managing infectious diseases are essential for nurses.

### Soft skills for nurses in epidemics

During epidemics, nurses are expected to have different competencies that can be categorized as soft skills in epidemics. These include leadership, organizational, communication, critical thinking, conflict management, problem solving, resilience, stress management, high internal motivations, accountability, adaptability, and creativity and artistic vision. For example, during recent pandemic general wards changed to COVID-19 wards that needed management and organizational skills. During COVID-19 pandemic, there were considerable shortage of the nurses, at the other hand nursing students, and ordinary people came to hospitals as volunteers that needed organizational competencies. The soft skills help nurses to manage chaotic situations, and communicate effectively with other professionals and stakeholders. The subcategories were internal and external professional communication, crises management and leadership, resilience and endurance, self-actualization, creativity and artistic vision.

Regarding leadership, a 28-year-old female nurse said: *“There was serious nursing shortage in the hospital, some nurses got sick, and some were so terrified that left the job. Some nursing students volunteered, and some nurses from ENT hospital transferred to this hospital. It was very important to manage this wide range of staff with different experiences, and more important, it was crucial that we as nurse support each other.”* Another participant stated: *“During the epidemic, the nurses and physicians became very close. We were all frightened, and didn’t know what to do. That look from up to dawn that physicians usually have disappeared. The communication was much better.”* Regarding crises management, one of the participants said: *“I was working at the pediatric ward, but when the hospital was loaded with COVID patients, they brought adult patients to our ward, but we had no basic equipment for the adults. We didn’t have any adult ambo bags. They had no plans. I even remember that the first day that we were admitting the adult patients, they started to change the pediatric beds with adult beds.”*

Nurses with higher resilience, courage, accountability, and altruism were more successful in working during epidemics. A participants stated: *“One of my colleagues who was really successful in caring for patients, was kind and passionate, and you could trust her. During the time of Corona, I saw her several times on the emergency ward transferring patients with wheelchairs and feeding them, at the time when even families were frightened and didn’t come to visit. She devoted herself to the patients.”* Another participant said: *“My wife was infected with COVID, my 13-year-old son was infected twice, but I never left my work even for a day. At that time, all we could think about was our patients, and we could not think about ourselves. However, every patient is a family’s father, someone’s child.”*

## Discussion

**Fig. 1:**
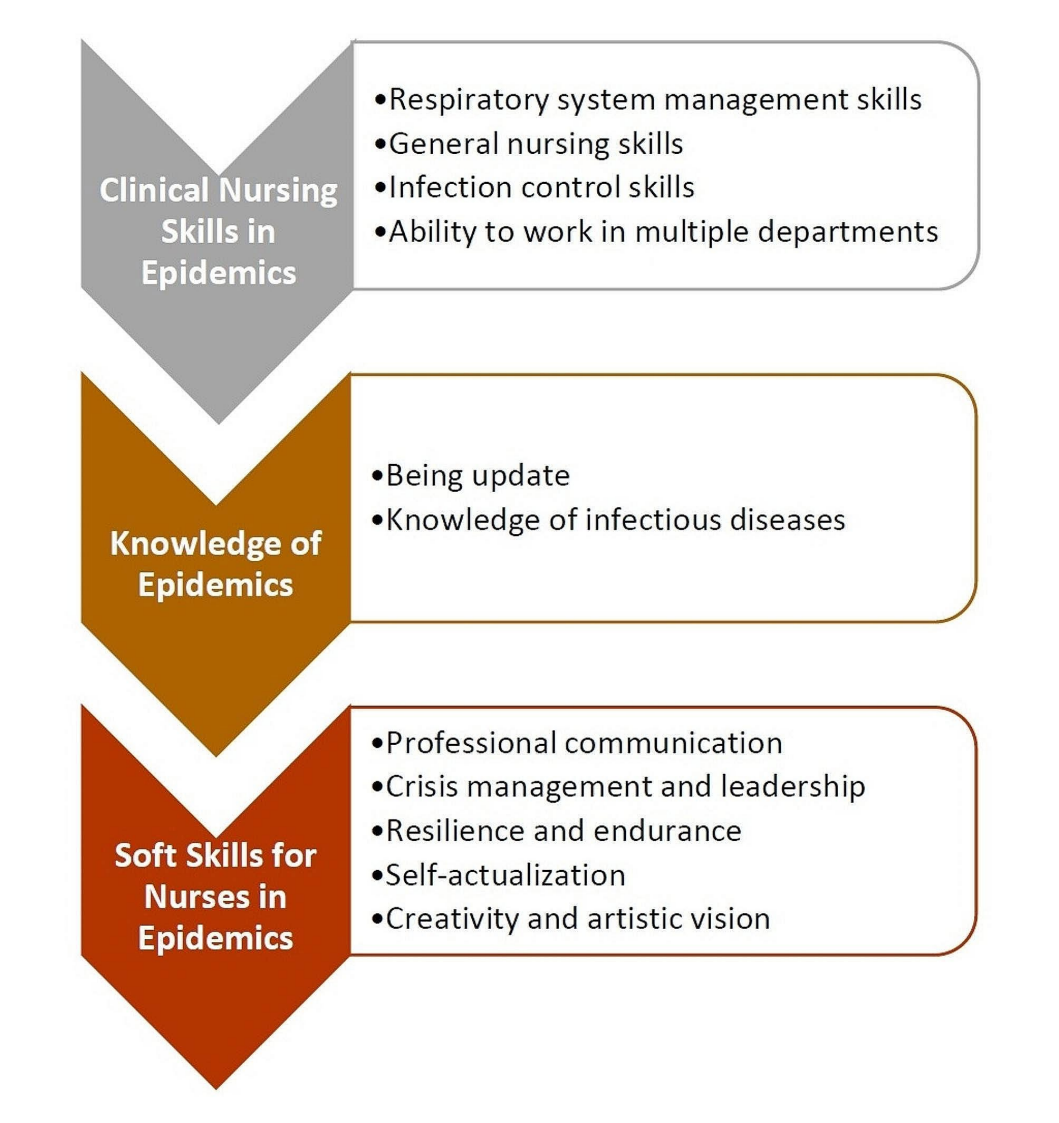
The illustration of nursing competencies in epidemics

The present study was conducted to determine different aspects of nursing clinical competency during epidemics, by reviewing the experiences of nurses during COVID-19 pandemic. The clinical competencies in epidemics can be categorized into 3 dimensions of clinical nursing skills in epidemics, knowledge of epidemics, and soft skills for nurses in epidemics. The illustration of the dimensions can be seen in Fig. 1.

A scoping review has defined 5 common domains of nurses’ competencies in public health emergencies as communication skills, self-protection skills, basic knowledge of a public health emergency, laws and ethics and the capacity for organizational collaboration [16]. A Delphi study in China described the infectious disease emergency response competencies for nurses in three domains of knowledge (infectious disease management and occupational protection), attitudes (psychological traits, and professional attitude), and skills (nursing practice, hospital infection management, education, and communication) [17]. Some domains of these studies show consistency with current study.

Nurses should have the clinical and technical skills to address the caring needs of the patients. A review study showed that there were 17 respiratory guidelines for nurses, that most of them were related to asthma, COPD, and lung cancer. There were 3 guidelines for pneumonia, 2 for Influenza, and 1 for tuberculosis. Only tuberculosis guideline received a high quality rate according to AGREE II criteria [18]. The respiratory skill guidelines are essential for education and skill development training for nurses to reduce any inconsistencies in the delivery of bedside care to the patient. Nurses should have opportunities to update their knowledge to maintain their ability to carry out technical skills. There are wide range of required nursing skills that don’t have clear or evidence based guidelines.

During epidemics, there is a high risk of respiratory deterioration, and many patients require endotracheal intubation. This technique is done by physicians, or practitioners specifically trained in airway management. Nurses often assist with the procedure [19]. According to the participants many patients needed intubation during COVID-19 pandemic, when only nurses were available in the ward. It seems more nurses need special training for intubation, and other necessary skills to be prepared for epidemics.

A study in UK showed that from nursing education institutions, just over a third spent over 4 h on respiratory pharmacology, local and national respiratory guidelines and information on pulmonary rehabilitation and other interventions for the management of respiratory conditions [20]. It seems that training of respiratory management skills need improvement.

Participants mentioned infection control skills as important competencies for nurses during epidemics. A study in Australia and Taiwan showed that experts identified essential infection control competencies for nurses as: hand hygiene, personal protective equipment, standard precautions and additional precautions, basic microbiology, waste management, cleaning, disinfection and sterilization skills. The majority of experts (75.4%) agreed that infection control competency levels of newly graduated nurses were inadequate [21]. Nurses were the first people to provide care for patients and prevent the transmission of COVID-19 in the hospitals and social environments. Therefore, nurses’ preparation for epidemics is necessary and includes proper use of personal protective equipment to protect themselves. Nurses need to be familiar with infection control guidelines, methods of quarantine, and preventive measures in healthcare environments [1]. The competencies related to infection control need to be strengthen in nursing curriculum and continuous education.

During epidemics nurses should have a competency to learn, and use update knowledge about the disease and its management. A study in Iran showed that nurses sources of information regarding COVID-19 were the Ministry of Health’s and World Health Organization’s websites (51.2%) followed by social media networks (25.1%), friends and colleagues (12.6%) and online courses (11.1%). This study showed that the majority of nurses had adequate information about the nature of the virus, the most common manifestations, incubation period, and its transmissions. Just half of the nurses answered correctly that most people recover from COVID-19 without the need for special treatment [22]. In Singapore nurses possessed moderate levels of knowledge about sepsis and confidence in recognizing and responding to patients with sepsis. Only 52.0% could correctly define sepsis [23]. These study shows that knowledge of nurses in some areas of infectious diseases is not adequate.

The collection of competencies such as leadership, resilience, creativity, and self-actualization, that was labeled as soft skills in current study was essential for nurses to work effectively during pandemics. A study showed that although nurses had good levels of competency during COVID-19 pandemic, but the level of their psychological ability was moderate [24]. . Another study revealed that nurses’ work stress, occupational burnout and anxiety during the COVID-19 was considerable and could negatively affect the physical and mental health of the novice nurses [25]. Therefore, competencies in stress management, and ability to work in difficult conditions need improvement.

A review showed that nurses play different roles in crises that need the combination of knowledge, perception and judgement, with cognitive, psychomotor and interpersonal skills [26]. According to the results of this study, to improve the quality of care for the patients, nurses should be aware of their roles in crises. In a cross-sectional study about the nurses’ perception of the main competencies in disasters (2020), it was revealed that it is necessary to merge disaster management education into the educational curriculum of nursing [27]. The abilities of crises management and triage, were in line with the present study.

Papadopoulos in 2020, mentioned the concept of cultural competency during the COVID-19 epidemic. The importance of correlation, especially during hard times, has been emphasized. This correlation has helped people to overcome the destructive effects of the pandemic. Therefore, it is recommended to prepare nurses for overcoming the disasters trough cultural competencies and provision of compassionate, and spiritual support [28]. The world was not prepared for the COVID-19 pandemic. Getting prepared for the next great global health disaster should start from now. Nurses have the potential to prevent mortality and suffering, and they are effective member of the healthcare team during epidemics, so they should be prepared adequately for their expected roles. The nursing competencies have been discussed extensively, but there are few studies focusing on the competencies of nurses during epidemics, using the lived experiences of the nurses. We tried to provide the detailed information about the required competencies according to the experiences of the nurses during COVID-19 crisis. This might be the strength of the study.

### Implications of the study

According to the findings, the following suggestions are recommended to nursing managers:

- Curriculum of nursing education needs improvement in subjects of infection control, isolation, sterilization, disinfection, and respiratory management skills.
- More nurses need special training in intubation, respiratory physiotherapy, managing patients with ECMO, and working with ventilators.
- It is recommended that nurses spend periodical rotations in emergency departments, and ICUs to be prepared for possible epidemics.
- There is a need for developing evidence based guidelines, and update information about infectious disease managements, and crisis management.
- Nursing managers, and professional institutes can develop programs to improve soft skills such as stress management, leadership, communication, resilience, and creativity in nurses.
- Clinical nurses’ competencies in epidemics need to be evaluated intermittently.

### Limitations

One of the limitations of the present study was that only experiences of hospital nurses were obtained, and the experiences of community health nurses, and patients were not considered. Therefore, a study about the nursing competencies with participation of community health nurses and patients can complete the current study.

## Conclusion

Three domains of clinical nursing skills, knowledge of epidemics, and soft skills for nurses in epidemics were defined as required competencies in this study. Healthcare authorities should take effective steps toward institutionalizing the required clinical competencies and skills, especially during the time of epidemics. We suggest audits about the effectiveness of interventional programs for improving competencies in nurses. The development of standard scales for measuring the nursing competencies in epidemics also is recommended for future studies.

### Electronic supplementary material

Below is the link to the electronic supplementary material.

Supplementary Material 1

## Data Availability

The datasets used and/or analyzed during the current study are available from the corresponding author on reasonable request.
