# Supplementary material for: Clinical nursing competency during epidemics: a qualitative content analysis
Source: BMC Nurs. 2024 May 3;23:306. doi: 10.1186/s12912-024-01977-y (PMC11071148; doi:10.1186/s12912-024-01977-y)
Supplement: Supplementary file 1 — Supplementary Material 1 [file 12912_2024_1977_MOESM1_ESM.docx]

The interview used in the study was developed for this study and has not been published elsewhere. The semi-structured interviews were conducted with 12 nurses.

Interview started with the general question of “explain one of your work days during the COVID-19 pandemic”, and continued with more specific questions such as “what would have helped you during the pandemic to provide better care for patients? What would have caused problem for you during the pandemic to provide care for the patients? Explain your experiences of caring for patients during the pandemic? And which nurses with which characteristics were more successful in caring for the patients during the pandemic?”. The Sampling was continued until data saturation.
